# 1794. Analysis of Antimicrobial Resistance Patterns in a Large Sample of US Hospitals Before and After COVID-19

**DOI:** 10.1093/ofid/ofad500.1623

**Published:** 2023-11-27

**Authors:** Tarjani M Ranade, Meggie Griffin, Rebecca Schwei, Michael Pulia

**Affiliations:** University of Wisconsin - Madison, Madison, Wisconsin; University of Wisconsin - Madison, Madison, Wisconsin; University of Wisconsin-Madison, Madison, Wisconsin; University of Wisconsin-Madison, Madison, Wisconsin

## Abstract

**Background:**

Significant concerns have been raised about a potential acceleration of trends in antimicrobial resistance (AMR) due to the COVID-19 pandemic. The pandemic disrupted infection control and antimicrobial stewardship processes and introduced a novel respiratory infection that mimics bacterial pneumonia, resulting in increases in hospital acquired infections and overall antibiotic prescribing. The objective of this study is to compare AMR of key bacterial pathogens before and during the pandemic to identify any concerning trends.

**Methods:**

We used bacterial culture data (blood, respiratory, urine, and wound) from 313 U.S. hospitals in the Premier Healthcare Database to compare AMR patterns pre-COVID and during-COVID, defined as Jan. 2019 to Feb. 2020 and Mar. 2020 to Mar. 2022, respectively. We characterized % resistant cultures for three organisms identified as major threats to public health: methicillin-resistant *Staphylococcus aureus* (MRSA), vancomycin-resistant *Enterococcus* (VRE) and extended-spectrum beta-lactamase (ESBL). In addition to descriptive results, we assessed for statistically significant changes between pre and during-COVID using chi-square tests.

**Results:**

There were 141,027 cultures analyzed for MRSA, 104,770 for VRE and 655,240 for ESBL. Wound was the most common culture type for MRSA (51.20%), while urine was most common for VRE and ESBL (75.84% and 86.21%, respectively). Between pre and during-COVID, the percent of MRSA isolates decreased (48.74% to 46.66% , p < 0.001), VRE isolates increased (11.45% to 12.06%, p = 0.004), and ESBL infections decreased (8.22% to 8.04%, p = 0.009) (Table 1). There were differences in AMR across all pathogens by race (p < 0.001) and ethnicity (p < 0.001) both pre and during-COVID. Risk of AMR infections increased among Black and Hispanic patients during COVID (Table 2).

**Figure:**
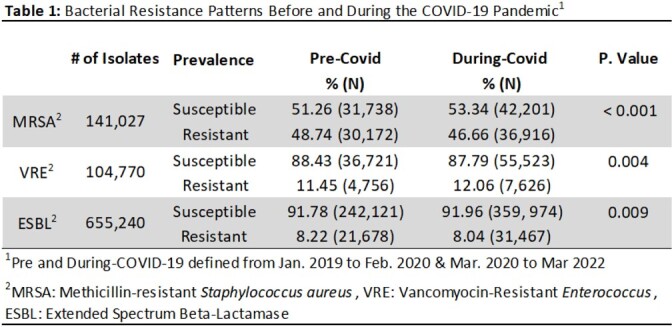

**Figure:**
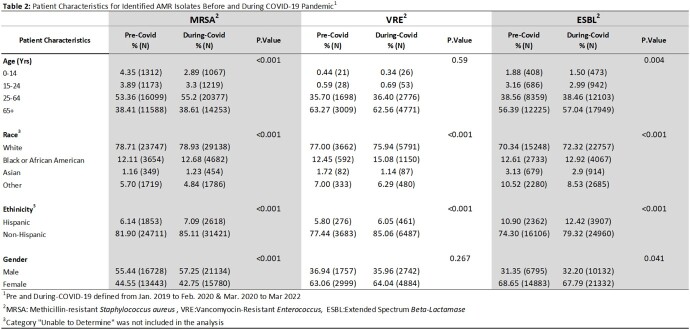

**Conclusion:**

Unexpectedly, we observed a statistically significant decrease in AMR for MRSA and ESBL during-COVID. AMR risk among racial and ethnic minority groups further increased during-COVID, raising concern about this as an additional area of disparity. The clinical significance of these results remains unclear and further research is needed to characterize the short and long term impact of COVID on AMR.

**Disclosures:**

**All Authors**: No reported disclosures

